# Books and Pamphlets Received

**Published:** 1887-05

**Authors:** 


### BOOKS AND PAMPHLETS RECEIVED.

*Manual of Treatment;* a Concise Presentation of the Modern Methods of Treating Disease employed by the best Authors, Teachers, and Practitioners. Arranged with special reference to the needs of American Practitioners. By C. F. Taylor, M. D., Editor of The Medical World, and W. F. Waugh, A. M., M. D., Professor of Practice and Clinical Medicine in the Medico-Chirurgical College of Philadelphia; Physician to the Medico-Chirurgical Hospital of Philadelphia ; Member of the American Academy of Medicine ; of the Academy of Natural Science ; of Microscopic and Biological Section, etc. Published by the Medical World, 1520 Chestnut street, Philadelphia.

This is a work of over five hundred pages, in which the authors have sought to condense, in clear and ready shape, the modern methods of treating disease. It is well and ably written, and must prove most acceptable to the busy practitioner who, in the press and hurry of professional labor, desires to post himself in the emergencies that almost daily arise. Here he finds what is regarded the latest and most approved remedies and methods of treatment. The book is of convenient size and, being alphabetically arranged, is very handy for reference. We commend it to our readers.

*Diseases of the Digestive Organs in Infancy and Childhood;* with chapters on the Investigation of Disease and on the general Management of Children. By Louis Starr, M. D., Clinical Professor of Diseases of Children in the Hospital of the University of Pennsylvania; Physician to the Children's Hospital, etc. With colored plate and other illustrations. Philadelphia, Pa.: P. Blakiston, Son & Co. 1886.

This is a useful and practical work of 383 octavo pages. The chapter on the investigation of disease with which the book opens is especially useful to the young and inexperienced practitioner. We are particularly impressed with the remarks on Respiration, Pulse, and Temperature. The remarks upon the Mouth and Fauces are also instructive and interesting. In the habitual constipation of children we note that he objects to castor oil and rhubarb as being followed after their action by a secondary binding effect. Among other useful formulæ advised, we copy the following :

℞. Mannæ opt.................................................

Magnesia carb, aa.......................................... ʒ ij,

Ext. sennæ, fl.............................................. ℥ ss,

Syrupi.......................................................... ℥ j,

Aquæ menth to make...................................... ℥ iv. M.

Dose, a teaspoonful one to three times per day for a child 6 months old.

*Diagnosis of Diseases of the Brain and of the Spinal Cord.* By W. R. Gowers, M. D., F. R. C. P., Assistant Professor of Clinical Medicine in University College; Physician to University College Hospital, and to the National Hospital for the Paralyzed and Epilectic. New York : Wm. Wood & Co. 1885.

An ably written book of 293 octavo pages, made up of a series of lectures delivered at University College Hospital. First, the medical anatomy is considered ; next, the symptoms of brain disease, hemiplegia, convulsions, etc. ; sensory symptoms, cranial nerve symptoms, mental symptoms, affections of speech; ophthalmoscopic changes ; local diagnosis and localization ; pathological diagnosis ; diseases of the cord, medical anatomy of, physiology of, etc. ; destruction of functional and organic disease. Plates, with illustrative diagrams, etc.

*Mental Pathology and Therapeutics.* By W. Griesenger, M. D., Professor of Clinical Medicine and Mental Science in the University of Berlin ; Honorary Member of the Medico-Psycological Association, etc. Translated from the German (second edition) by C. Lockhart Robertson, M. D , Cantabury, Medical Superintendent of the Sussex Asylum, Hayward's Heath, and James Rutherford, M. D., Edinburgh. New York : William Wood & Co.

The work contains 375 octavo pages, and will be welcomed by all lovers of mental science, as "Professor Griesinger is essentially the representative and the acknowledged leader of the modern German school of medical psycology." Every form of mental disease is ably discussed in the book—as of mental depression, hypochondriacs, mania, monomania, idiocy, dementia, cretenism, etc. The principles and facts of treatment of mental disorders as practiced in Germany are especially instructive to all in this country who are in any way concerned in lunatic asylums, etc.

*Insanity and its Treatment.* Lectures on the treatment, medical and legal, of insane patients. By G. Fielding Blandford, M. D., Oxon, Fellow of the Royal College of Physicians, London ; late Lecturer on Psycological Medicine at the School of St. George's Hospital, London. Third edition. Together with Types of Insanity, an illustrated guide in the physical diagnosis of mental diseases. By Allan McLane Hamilton, M. D., one of the Consulting Physicians to the Insane Asylums of New York City, and the Hudson River State Hospital for the Insane, etc. New York : William Wood & Co. 1886.

Three hundred and seventy-nine large octavo pages, well printed in plain type, and neatly bound in red cloth. Treats ably of the phenomena of Mind, the pathology of insanity, morbid appearance of meninges, etc. ; cases of insanity ; symptoms ; acts of the insane ; the two extremes of insanity ; acute delirious mania ; general paralysis of the insane ; insanity without delusions ; impulsive insanity ; examination of patients ; treatment, etc. It is a very recent and a very instructive work.

*Analysis of the Urine,* with special reference to the Diseases of the Genito-Urinary Organs. By R. B. Hoffman, Professor in the University of Gratz, and R. Ultzmann, Docent in the University of Vienna. Translated by T. Barton Brum, A. M., M. D.. late professor of the Practice of Medicine in the Baltimore Polyclinic and Post-Graduate Medical School, and Lecturer in Chemical Medicine in the University of Medicine, and H. Holbrook Curtis, Ph. B., M. D., Fellow of the New York Academy of Medicine ; Member of the County Society, etc. Second edition, revised and enlarged. New York: D. Appleton & Co. 1886.

It is affirmed of the work that, while not pretending to be an elaborate and exhaustive treatise on the diseases of the genito-urinary organs, it nevertheless contains more valuable and practical suggestions on the subject than any other work in this line now extant. It is exceedingly popular in Germany, and must prove very acceptable in this country.

